# Supplementary material for: Vitamin D Receptor Genetic Variation and Cancer Biomarkers among Breast Cancer Patients Supplemented with Vitamin D3: A Single-Arm Non-Randomized Before and After Trial
Source: Nutrients. 2019 Jun 4;11(6):1264. doi: 10.3390/nu11061264 (PMC6628022; doi:10.3390/nu11061264)
Supplement: Supplementary file 1 [file nutrients-11-01264-s001.pdf]

Figure S1. Participants flowchart

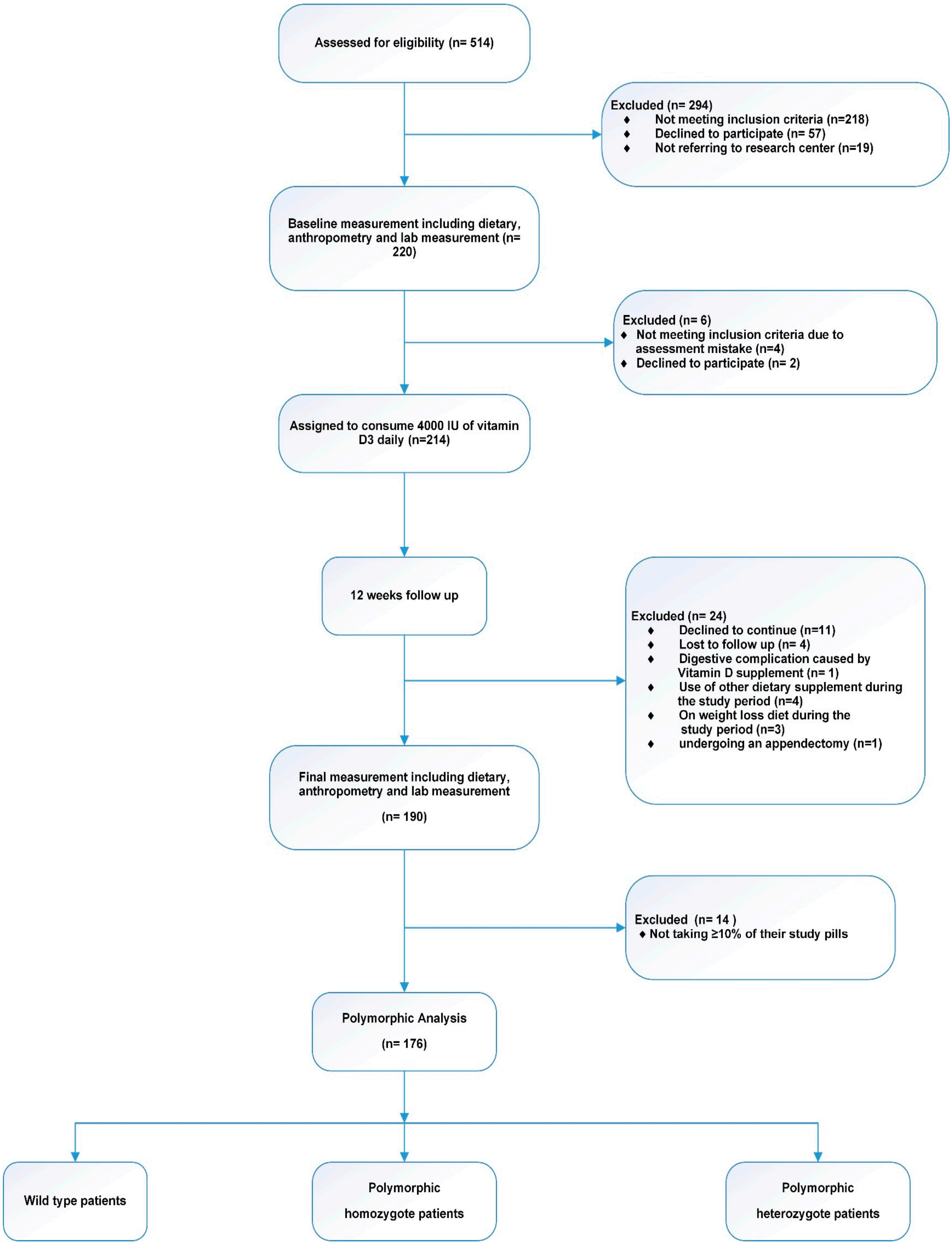

Figure S2. Graphical representation of changes in response variables before and after vitamin D3 supplementation (4000 IU/day) for 12 weeks.

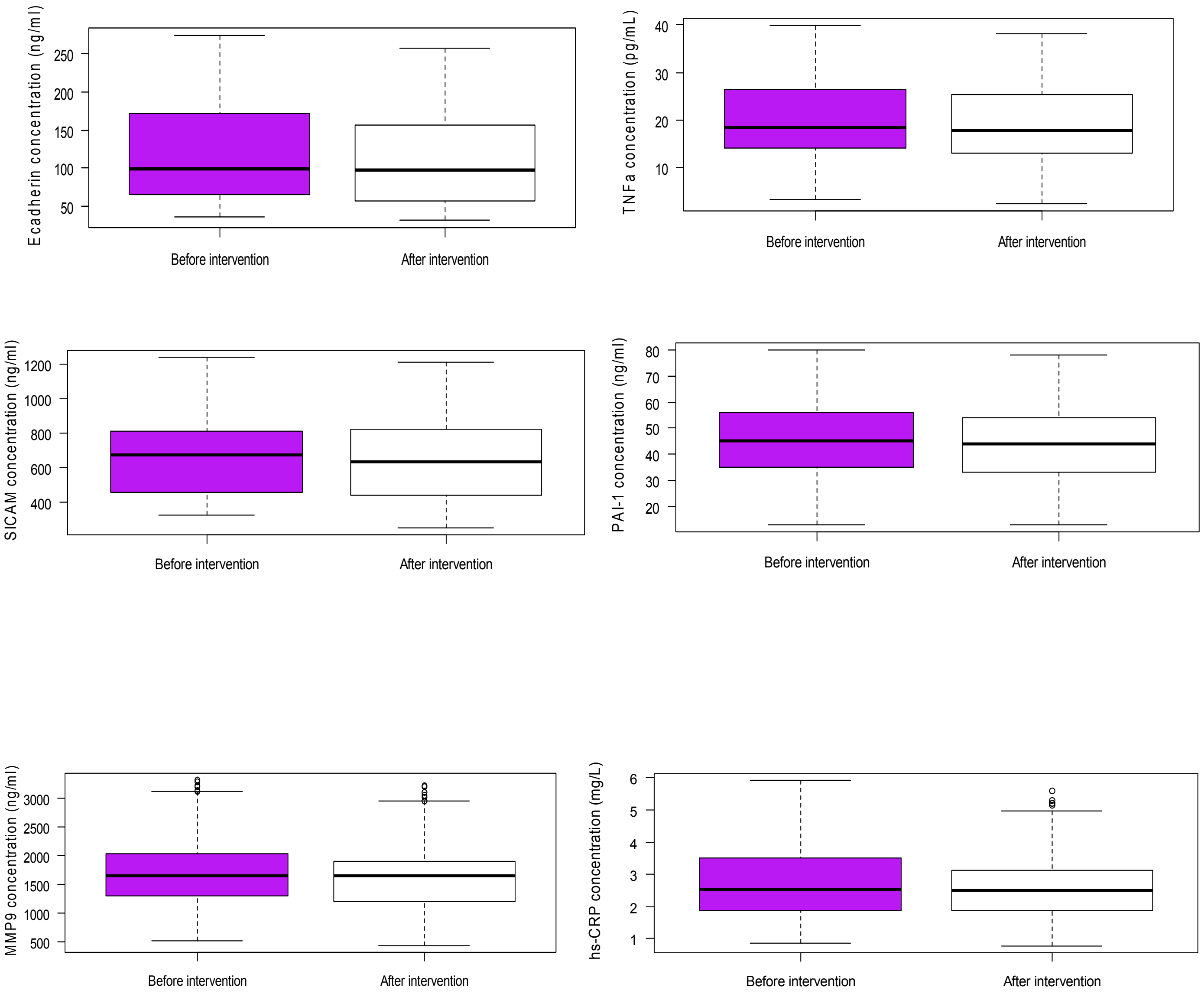

MMP9, matrix metallopeptidase 9;

SICAM-1, soluble intercellular adhesion molecule-1; TNFα, tumor necrosis factorα; PAI-1, plasminogen activator inhibitor-1, hs-CRP, human high sensitivity C-reactive protein.

Table S1. Haplo.Score analysis of matrix metallopeptidase 9 (MMP9) changes after vitamin D supplementation (4000 IU/day) for 12 weeks

**Haplotypes**						**Hap-Freq**	**Hap-Score**	**P value***
Cdx-2 FokI BsmI ApaI TaqI	Cdx2	*FokI*	*BsmI*	*ApaI*	*TaqI*			<0.001
1	G	f	b	A	t	0.021	-2.97	0.002
2	G	F	b	a	T	0.045	-2.05	0.03
3	G	f	b	a	T	0.023	-1.93	0.05
4	G	f	b	A	T	0.032	-1.81	0.06
5	G	f	B	a	T	0.044	-1.65	0.09
6	G	f	B	A	T	0.058	-1.27	0.20
7	G	f	B	A	t	0.080	-1.12	0.25
8	G	F	B	A	t	0.063	-1.10	0.26
9	G	F	B	A	T	0.139	-0.77	0.43
10	G	F	b	A	T	0.059	-0.63	0.52
11	G	F	B	a	t	0.038	-0.59	0.55
12	G	F	b	A	t	0.065	-0.34	0.72
13	G	F	B	a	T	0.059	-0.33	0.74
14	A	f	B	a	T	0.022	0.93	0.35
15	A	F	b	a	T	0.023	1.30	0.19
16	A	F	B	a	T	0.041	2.09	0.035
17	A	f	b	A	T	0.027	2.11	0.03
18	A	F	B	A	t	0.034	4.03	<0.001
19	A	F	B	A	T	0.073	4.31	<0.001
Cdx2 FokI BsmI								0.04
1	G	f	b	-	-	0.097	-8.944	<0.001
2	G	F	b	-	-	0.16	-2.223	0.02
3	G	f	B	-	-	0.188	-1.571	0.11
4	G	F	B	-	-	0.303	-0.424	0.67
5	A	f	b	-	-	0.021	0.785	0.43
6	A	f	B	-	-	0.021	1.105	0.26
7	A	F	b	-	-	0.044	1.748	0.08

8	A	F	B	-	-	0.162	3.483	<0.001
FokI TaqI Cdx2								<0.001
1	G	f	-	-	T	0.17	-2.78	0.005
2	G	f	-	-	t	0.10	-2.66	0.007
3	G	F	-	-	T	0.28	-2.13	0.03
4	G	F	-	-	t	0.17	-1.05	0.29
5	A	f	-	-	T	0.04	2.36	0.01
6	A	F	-	-	t	0.06	4.82	<0.001
7	A	F	-	-	T	0.14	5.10	<0.001

* P values were adjusted for age, baseline 25-hydroxy vitamin D (25(OH)D) and body mass index(BMI) and resulted from the false-discovery rate (FDR) methods.
Hap-Freq indicates the estimated frequency of the haplotype in the pool of all subjects.
